# 5-(4-Fluoro­phen­yl)-3-[5-methyl-1-(4-methyl­phen­yl)-1*H*-1,2,3-triazol-4-yl]-*N*-phenyl-4,5-dihydro-1*H*-pyrazole-1-carbothio­amide

**DOI:** 10.1107/S1600536813008155

**Published:** 2013-03-28

**Authors:** Bakr F. Abdel-Wahab, Seik Weng Ng, Edward R. T. Tiekink

**Affiliations:** a Applied Organic Chemistry Department, National Research Centre, Dokki, 12622 Giza, Egypt; b Department of Chemistry, University of Malaya, 50603 Kuala Lumpur, Malaysia; c Chemistry Department, Faculty of Science, King Abdulaziz University, PO Box 80203 Jeddah, Saudi Arabia

## Abstract

In the title compound, C_26_H_23_FN_6_S, the pyrazole ring has an envelope conformation, with the methine C atom being the flap atom. The thio­urea group is close to being coplanar with the pyrazole N atoms [N—N—C—S torsion angle = 176.78 (15)°], which allows for an intra­molecular N—H⋯N hydrogen bond; the connected triazole ring is nearly coplanar with this ring [N—C—C—N = −172.65 (19)°]. There is a significant twist between the pyrazole ring and attached fluoro­benzene ring [N—C—C—C = −18.8 (3)°] and a greater twist between triazole and attached tolyl ring [dihedral angle = 58.25 (14)°]. In the crystal, supra­molecular chains aligned along [40,10] are consolidated by π–π inter­actions between the triazole and phenyl rings [centroid–centroid distance = 3.7053 (13) Å].

## Related literature

For the biological activity and synthesis of related compounds, see: Abdel-Wahab, Abdel-Latif *et al.* (2012 ▶). For a related structure, see: Abdel-Wahab, Mohamed *et al.* (2012 ▶).
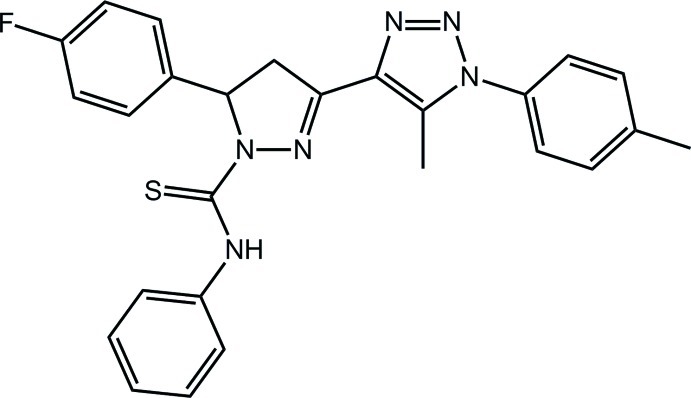


## Experimental

### 

#### Crystal data

C_26_H_23_FN_6_S
*M* *_r_* = 470.56
Monoclinic, 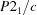

*a* = 6.5449 (5) Å
*b* = 26.1030 (17) Å
*c* = 14.3818 (8) Å
β = 100.604 (7)°
*V* = 2415.0 (3) Å^3^
*Z* = 4
Mo *K*α radiation
μ = 0.17 mm^−1^
*T* = 295 K
0.40 × 0.30 × 0.20 mm

#### Data collection

Agilent SuperNova Dual diffractometer with an Atlas detector
Absorption correction: multi-scan (*CrysAlis PRO*; Agilent, 2011 ▶) *T*_min_ = 0.802, *T*_max_ = 1.000
15173 measured reflections
5578 independent reflections
3313 reflections with *I* > 2σ(*I*)
*R*_int_ = 0.040

#### Refinement

*R*[*F* ^2^ > 2σ(*F* ^2^)] = 0.056
*wR*(*F* ^2^) = 0.162
*S* = 1.03
5578 reflections
313 parameters
H atoms treated by a mixture of independent and constrained refinement
Δρ_max_ = 0.17 e Å^−3^
Δρ_min_ = −0.21 e Å^−3^

### 

Data collection: *CrysAlis PRO* (Agilent, 2011 ▶); cell refinement: *CrysAlis PRO*; data reduction: *CrysAlis PRO*; program(s) used to solve structure: *SHELXS97* (Sheldrick, 2008 ▶); program(s) used to refine structure: *SHELXL97* (Sheldrick, 2008 ▶); molecular graphics: *ORTEP-3 for Windows* (Farrugia, 2012 ▶) and *DIAMOND* (Brandenburg, 2006 ▶); software used to prepare material for publication: *publCIF* (Westrip, 2010 ▶).

## Supplementary Material

Crystal structure: contains datablock(s) global, I. DOI: 10.1107/S1600536813008155/hg5303sup1.cif

Structure factors: contains datablock(s) I. DOI: 10.1107/S1600536813008155/hg5303Isup2.hkl

Supplementary material file. DOI: 10.1107/S1600536813008155/hg5303Isup3.cml

Additional supplementary materials:  crystallographic information; 3D view; checkCIF report

## Figures and Tables

**Table 1:** Hydrogen-bond geometry (Å, °)

*D*—H⋯*A*	*D*—H	H⋯*A*	*D*⋯*A*	*D*—H⋯*A*
N1—H3⋯N3	0.83 (3)	2.05 (3)	2.568 (3)	120 (2)

**Fig. 1.**
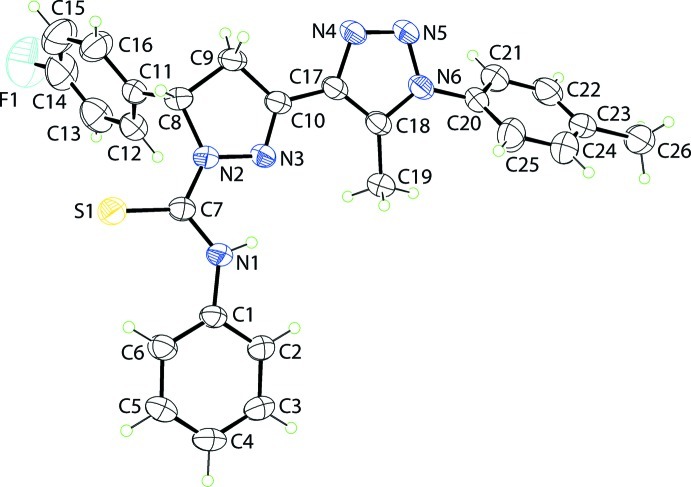
The molecular structure of (I) showing the atom-labelling scheme and displacement ellipsoids at the 35% probability level.

**Fig. 2.**
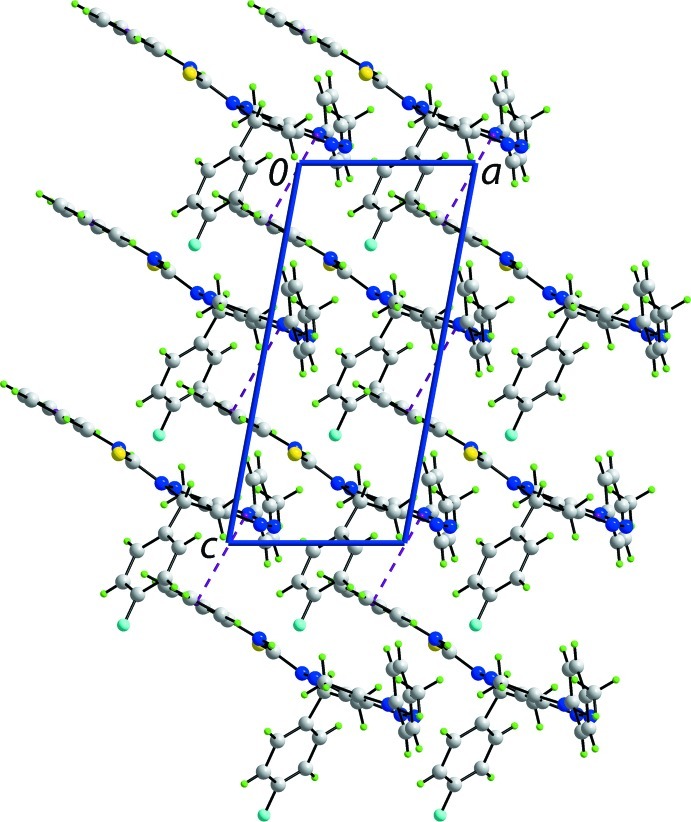
A view of the crystal packing in (I) highlighting the supramolecular chains sustained by π—π interactions (purple dashed lines).

**Fig. 3.**
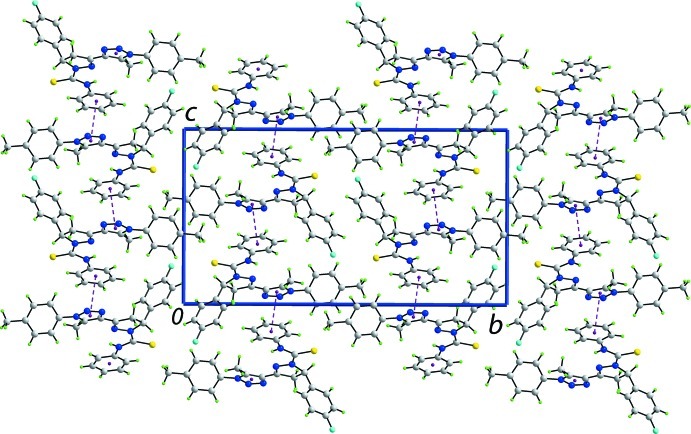
A view of the crystal packing in projection down the a axis. The π—π interactions are shown as purple dashed lines.

## supplementary crystallographic information

### Comment

In connection with studies into the biological studies on related pyrazolines (Abdel-Wahab, Abdel-Latif *et al.*, 2012), the title compound, (I), was investigated.

In (I), Fig. 1, the pyrazole ring has an envelope conformation with the methine-C8 atom being the flap atom. The thiourea group is close to co-planar with the N atoms of this ring [the N3—N2—C7—S1 torsion angle = 176.78 (15)°], which allows for an intramolecular N1—H···N3 hydrogen bond, Table 1, and the connected triazole ring is slightly twisted out of the plane through this ring [N3—C10—C17—N4 is -172.65 (19)°]. There is a significant twist between the pyrazole ring and attached fluorobenzene ring as seen in the N2—C8—C11—C12 torsion angle of -18.8 (3)°, and an even greater twist between triazole and attached tolyl ring with the dihedral angle being 58.25 (14)°. The relative dispositions of the terminal substituent in (I) resembles those found in a recently determined structure with pyrazole-*p*-tolyl and triazole-4-(piperidin-1-yl)phenyl substituents (Abdel-Wahab, Mohamed *et al.*, 2012).

The most prominent feature of the crystal packing is the formation of π—π interactions between the triazole and phenyl rings [inter-centroid distance = 3.7053 (13) Å, angle of inclination = 10.17 (12)° for symmetry operation *i*: 1 + *x*, 3/2 - *y*, 1/2 + *z*]. These lead to a supramolecular chains, aligned approximately along [1 0 2], and which aggregate in the *ac* plane with no specific interactions between them, Fig. 2. Layers thus formed stack along the *b* axis, Fig. 3.

### Experimental

The title compound was prepared according to the reported method (Abdel-Wahab, Abdel-Latif *et al.*, 2012). Colourless crystals were obtained from its DMF solution by slow evaporation at room temperature.

### Refinement

Carbon-bound H-atoms were placed in calculated positions (C—H = 0.93 to 0.98 Å) and were included in the refinement in the riding model approximation, with *U*_iso_(H) = 1.2–1.5*U*_equiv_(C). The nitrogen-bound H-atom was refined freely.

### Crystal data

**Table:**

C_26_H_23_FN_6_S	*F*(000) = 984
*M**_r_* = 470.56	*D*_x_ = 1.294 Mg m^−^^3^
Monoclinic, *P*2_1_/*c*	Mo *K*α radiation, λ = 0.71073 Å
Hall symbol: -P 2ybc	Cell parameters from 2937 reflections
*a* = 6.5449 (5) Å	θ = 2.9–27.5°
*b* = 26.1030 (17) Å	µ = 0.17 mm^−^^1^
*c* = 14.3818 (8) Å	*T* = 295 K
β = 100.604 (7)°	Prism, colourless
*V* = 2415.0 (3) Å^3^	0.40 × 0.30 × 0.20 mm
*Z* = 4	

### Data collection

**Table:**

Agilent SuperNova Dual diffractometer with an Atlas detector	5578 independent reflections
Radiation source: SuperNova (Mo) X-ray Source	3313 reflections with *I* > 2σ(*I*)
Mirror monochromator	*R*_int_ = 0.040
Detector resolution: 10.4041 pixels mm^-1^	θ_max_ = 27.6°, θ_min_ = 2.9°
ω scan	*h* = −8→8
Absorption correction: multi-scan (*CrysAlis PRO*; Agilent, 2011)	*k* = −33→33
*T*_min_ = 0.802, *T*_max_ = 1.000	*l* = −18→18
15173 measured reflections	

### Refinement

**Table:**

Refinement on *F*^2^	Primary atom site location: structure-invariant direct methods
Least-squares matrix: full	Secondary atom site location: difference Fourier map
*R*[*F*^2^ > 2σ(*F*^2^)] = 0.056	Hydrogen site location: inferred from neighbouring sites
*wR*(*F*^2^) = 0.162	H atoms treated by a mixture of independent and constrained refinement
*S* = 1.03	*w* = 1/[σ^2^(*F*_o_^2^) + (0.0643*P*)^2^ + 0.3514*P*] where *P* = (*F*_o_^2^ + 2*F*_c_^2^)/3
5578 reflections	(Δ/σ)_max_ = 0.001
313 parameters	Δρ_max_ = 0.17 e Å^−^^3^
0 restraints	Δρ_min_ = −0.21 e Å^−^^3^

### Special details

**Table:**

Geometry. All e.s.d.'s (except the e.s.d. in the dihedral angle between two l.s. planes) are estimated using the full covariance matrix. The cell e.s.d.'s are taken into account individually in the estimation of e.s.d.'s in distances, angles and torsion angles; correlations between e.s.d.'s in cell parameters are only used when they are defined by crystal symmetry. An approximate (isotropic) treatment of cell e.s.d.'s is used for estimating e.s.d.'s involving l.s. planes.
Refinement. Refinement of *F*^2^ against ALL reflections. The weighted *R*-factor *wR* and goodness of fit *S* are based on *F*^2^, conventional *R*-factors *R* are based on *F*, with *F* set to zero for negative *F*^2^. The threshold expression of *F*^2^ > σ(*F*^2^) is used only for calculating *R*-factors(gt) *etc*. and is not relevant to the choice of reflections for refinement. *R*-factors based on *F*^2^ are statistically about twice as large as those based on *F*, and *R*- factors based on ALL data will be even larger.

### Fractional atomic coordinates and isotropic or equivalent isotropic displacement parameters (Å^2^)

**Table:**

	*x*	*y*	*z*	*U*_iso_*/*U*_eq_	
S1	0.28247 (11)	0.59566 (2)	0.26576 (4)	0.0660 (2)	
F1	0.4921 (5)	0.53780 (11)	0.71572 (17)	0.1882 (13)	
N1	0.2779 (3)	0.69941 (8)	0.25175 (14)	0.0581 (5)	
N2	0.5580 (3)	0.66351 (7)	0.34286 (12)	0.0538 (5)	
N3	0.6368 (3)	0.71314 (7)	0.35592 (12)	0.0519 (5)	
N4	1.1621 (3)	0.75227 (8)	0.45157 (14)	0.0624 (5)	
N5	1.2414 (3)	0.79826 (8)	0.45671 (15)	0.0662 (6)	
N6	1.0814 (3)	0.83112 (8)	0.42485 (12)	0.0584 (5)	
C1	0.0777 (3)	0.71154 (8)	0.20191 (14)	0.0503 (5)	
C2	0.0342 (4)	0.76336 (9)	0.18895 (15)	0.0580 (6)	
H2	0.1373	0.7873	0.2105	0.070*	
C3	−0.1592 (4)	0.77990 (10)	0.14473 (15)	0.0635 (7)	
H3A	−0.1860	0.8148	0.1371	0.076*	
C4	−0.3132 (4)	0.74477 (11)	0.11172 (15)	0.0655 (7)	
H4	−0.4442	0.7557	0.0819	0.079*	
C5	−0.2697 (4)	0.69365 (11)	0.12361 (16)	0.0659 (7)	
H5	−0.3729	0.6699	0.1012	0.079*	
C6	−0.0762 (4)	0.67637 (9)	0.16809 (15)	0.0608 (6)	
H6	−0.0500	0.6414	0.1751	0.073*	
C7	0.3712 (4)	0.65505 (8)	0.28600 (14)	0.0504 (5)	
C8	0.7040 (3)	0.62449 (9)	0.39009 (14)	0.0532 (6)	
H8	0.7241	0.5976	0.3451	0.064*	
C9	0.9037 (3)	0.65642 (9)	0.41746 (16)	0.0572 (6)	
H9A	0.9644	0.6520	0.4837	0.069*	
H9B	1.0056	0.6472	0.3792	0.069*	
C10	0.8281 (3)	0.70984 (9)	0.39768 (14)	0.0503 (5)	
C11	0.6339 (4)	0.60126 (9)	0.47522 (15)	0.0547 (6)	
C12	0.4899 (4)	0.62432 (10)	0.51996 (17)	0.0666 (7)	
H12	0.4242	0.6543	0.4956	0.080*	
C13	0.4429 (5)	0.60252 (14)	0.6021 (2)	0.0936 (10)	
H13	0.3470	0.6179	0.6336	0.112*	
C14	0.5405 (7)	0.55819 (17)	0.6353 (2)	0.1117 (13)	
C15	0.6779 (7)	0.53446 (15)	0.5924 (3)	0.1172 (13)	
H15	0.7404	0.5041	0.6164	0.141*	
C16	0.7249 (5)	0.55604 (11)	0.5117 (2)	0.0877 (9)	
H16	0.8202	0.5398	0.4810	0.105*	
C17	0.9534 (3)	0.75537 (9)	0.41700 (14)	0.0520 (5)	
C18	0.8996 (3)	0.80599 (9)	0.39990 (14)	0.0530 (5)	
C19	0.6961 (4)	0.83117 (10)	0.36692 (18)	0.0669 (7)	
H19A	0.7083	0.8672	0.3802	0.100*	
H19B	0.5940	0.8165	0.3991	0.100*	
H19C	0.6543	0.8261	0.3000	0.100*	
C20	1.1213 (4)	0.88469 (10)	0.41824 (18)	0.0636 (6)	
C21	1.2087 (4)	0.91154 (11)	0.4967 (2)	0.0790 (8)	
H21	1.2441	0.8953	0.5550	0.095*	
C22	1.2439 (5)	0.96392 (12)	0.4878 (3)	0.0979 (10)	
H22	1.3019	0.9826	0.5413	0.117*	
C23	1.1953 (5)	0.98862 (13)	0.4024 (3)	0.1023 (11)	
C24	1.1078 (6)	0.95997 (14)	0.3244 (3)	0.1113 (12)	
H24	1.0730	0.9758	0.2657	0.134*	
C25	1.0717 (5)	0.90862 (12)	0.3320 (2)	0.0926 (10)	
H25	1.0134	0.8899	0.2786	0.111*	
C26	1.2352 (5)	1.04560 (13)	0.3937 (4)	0.151 (2)	
H26A	1.2715	1.0604	0.4556	0.227*	
H26B	1.1121	1.0619	0.3599	0.227*	
H26C	1.3473	1.0506	0.3599	0.227*	
H3	0.355 (5)	0.7242 (10)	0.2690 (19)	0.080 (9)*	

### Atomic displacement parameters (Å^2^)

**Table:**

	*U*^11^	*U*^22^	*U*^33^	*U*^12^	*U*^13^	*U*^23^
S1	0.0670 (5)	0.0540 (4)	0.0691 (4)	0.0000 (3)	−0.0082 (3)	−0.0048 (3)
F1	0.231 (3)	0.219 (3)	0.1311 (18)	−0.018 (2)	0.0773 (19)	0.0845 (19)
N1	0.0465 (12)	0.0515 (12)	0.0686 (12)	−0.0016 (9)	−0.0094 (10)	0.0012 (10)
N2	0.0447 (11)	0.0541 (11)	0.0577 (10)	−0.0005 (9)	−0.0038 (9)	0.0055 (9)
N3	0.0419 (11)	0.0577 (11)	0.0533 (10)	−0.0040 (9)	0.0018 (9)	0.0021 (8)
N4	0.0391 (11)	0.0766 (14)	0.0691 (12)	−0.0062 (10)	0.0038 (9)	0.0110 (10)
N5	0.0392 (11)	0.0766 (14)	0.0799 (13)	−0.0071 (10)	0.0033 (10)	0.0126 (11)
N6	0.0420 (11)	0.0739 (13)	0.0585 (10)	−0.0079 (10)	0.0069 (9)	0.0072 (10)
C1	0.0435 (13)	0.0598 (14)	0.0438 (10)	0.0014 (10)	−0.0018 (9)	0.0020 (10)
C2	0.0570 (15)	0.0587 (14)	0.0543 (12)	0.0013 (11)	−0.0001 (11)	0.0060 (10)
C3	0.0608 (16)	0.0713 (16)	0.0552 (13)	0.0141 (13)	0.0023 (12)	0.0123 (12)
C4	0.0492 (15)	0.0914 (19)	0.0517 (12)	0.0119 (14)	−0.0018 (11)	0.0060 (13)
C5	0.0490 (15)	0.0826 (18)	0.0605 (13)	−0.0044 (13)	−0.0046 (12)	−0.0059 (13)
C6	0.0532 (15)	0.0620 (14)	0.0617 (13)	0.0002 (12)	−0.0042 (11)	−0.0070 (11)
C7	0.0473 (13)	0.0569 (13)	0.0445 (10)	0.0017 (10)	0.0020 (10)	0.0008 (10)
C8	0.0475 (13)	0.0581 (14)	0.0511 (11)	0.0086 (10)	0.0013 (10)	0.0023 (10)
C9	0.0400 (13)	0.0709 (15)	0.0592 (12)	0.0056 (11)	0.0049 (10)	0.0081 (11)
C10	0.0392 (12)	0.0660 (14)	0.0450 (10)	0.0006 (10)	0.0059 (9)	0.0041 (10)
C11	0.0489 (14)	0.0561 (13)	0.0555 (12)	−0.0058 (11)	−0.0001 (11)	0.0023 (10)
C12	0.0601 (16)	0.0752 (17)	0.0637 (14)	−0.0069 (13)	0.0092 (13)	−0.0015 (13)
C13	0.085 (2)	0.122 (3)	0.0793 (19)	−0.018 (2)	0.0293 (17)	−0.0031 (19)
C14	0.124 (3)	0.132 (3)	0.081 (2)	−0.026 (3)	0.023 (2)	0.041 (2)
C15	0.131 (3)	0.108 (3)	0.111 (3)	0.013 (2)	0.020 (3)	0.055 (2)
C16	0.092 (2)	0.082 (2)	0.0896 (19)	0.0178 (17)	0.0177 (17)	0.0252 (17)
C17	0.0385 (12)	0.0715 (15)	0.0453 (10)	−0.0048 (11)	0.0058 (9)	0.0062 (10)
C18	0.0407 (13)	0.0708 (15)	0.0479 (11)	−0.0051 (11)	0.0088 (10)	0.0043 (11)
C19	0.0452 (14)	0.0740 (17)	0.0788 (16)	0.0009 (12)	0.0045 (12)	0.0087 (13)
C20	0.0442 (14)	0.0697 (16)	0.0753 (16)	−0.0098 (12)	0.0070 (12)	0.0136 (13)
C21	0.0627 (18)	0.0807 (19)	0.0870 (18)	−0.0114 (14)	−0.0036 (15)	0.0070 (15)
C22	0.062 (2)	0.079 (2)	0.142 (3)	−0.0133 (16)	−0.009 (2)	−0.003 (2)
C23	0.0480 (17)	0.078 (2)	0.175 (3)	−0.0001 (15)	0.004 (2)	0.039 (2)
C24	0.088 (3)	0.112 (3)	0.129 (3)	−0.015 (2)	0.007 (2)	0.055 (2)
C25	0.096 (2)	0.097 (2)	0.0811 (18)	−0.0215 (18)	0.0057 (17)	0.0258 (17)
C26	0.076 (2)	0.074 (2)	0.291 (6)	−0.0018 (18)	−0.002 (3)	0.058 (3)

### Geometric parameters (Å, º)

**Table:**

S1—C7	1.662 (2)	C10—C17	1.442 (3)
F1—C14	1.363 (4)	C11—C12	1.374 (3)
N1—C7	1.358 (3)	C11—C16	1.381 (3)
N1—C1	1.409 (3)	C12—C13	1.395 (4)
N1—H3	0.83 (3)	C12—H12	0.9300
N2—C7	1.358 (3)	C13—C14	1.365 (5)
N2—N3	1.394 (2)	C13—H13	0.9300
N2—C8	1.474 (3)	C14—C15	1.333 (5)
N3—C10	1.288 (3)	C15—C16	1.375 (4)
N4—N5	1.305 (3)	C15—H15	0.9300
N4—C17	1.367 (3)	C16—H16	0.9300
N5—N6	1.366 (3)	C17—C18	1.378 (3)
N6—C18	1.348 (3)	C18—C19	1.482 (3)
N6—C20	1.429 (3)	C19—H19A	0.9600
C1—C6	1.383 (3)	C19—H19B	0.9600
C1—C2	1.388 (3)	C19—H19C	0.9600
C2—C3	1.377 (3)	C20—C21	1.361 (4)
C2—H2	0.9300	C20—C25	1.373 (4)
C3—C4	1.381 (4)	C21—C22	1.396 (4)
C3—H3A	0.9300	C21—H21	0.9300
C4—C5	1.368 (4)	C22—C23	1.372 (5)
C4—H4	0.9300	C22—H22	0.9300
C5—C6	1.385 (3)	C23—C24	1.382 (5)
C5—H5	0.9300	C23—C26	1.519 (4)
C6—H6	0.9300	C24—C25	1.369 (4)
C8—C11	1.511 (3)	C24—H24	0.9300
C8—C9	1.539 (3)	C25—H25	0.9300
C8—H8	0.9800	C26—H26A	0.9600
C9—C10	1.489 (3)	C26—H26B	0.9600
C9—H9A	0.9700	C26—H26C	0.9600
C9—H9B	0.9700		
			
C7—N1—C1	133.6 (2)	C11—C12—C13	119.7 (3)
C7—N1—H3	110 (2)	C11—C12—H12	120.2
C1—N1—H3	116 (2)	C13—C12—H12	120.2
C7—N2—N3	120.12 (17)	C14—C13—C12	118.7 (3)
C7—N2—C8	126.89 (18)	C14—C13—H13	120.7
N3—N2—C8	112.83 (17)	C12—C13—H13	120.7
C10—N3—N2	107.71 (17)	C15—C14—F1	119.6 (4)
N5—N4—C17	109.1 (2)	C15—C14—C13	123.0 (3)
N4—N5—N6	106.63 (18)	F1—C14—C13	117.5 (4)
C18—N6—N5	111.6 (2)	C14—C15—C16	118.4 (3)
C18—N6—C20	128.4 (2)	C14—C15—H15	120.8
N5—N6—C20	119.91 (19)	C16—C15—H15	120.8
C6—C1—C2	118.8 (2)	C15—C16—C11	121.5 (3)
C6—C1—N1	125.3 (2)	C15—C16—H16	119.2
C2—C1—N1	115.9 (2)	C11—C16—H16	119.2
C3—C2—C1	121.1 (2)	N4—C17—C18	109.1 (2)
C3—C2—H2	119.5	N4—C17—C10	121.1 (2)
C1—C2—H2	119.5	C18—C17—C10	129.7 (2)
C2—C3—C4	120.1 (2)	N6—C18—C17	103.6 (2)
C2—C3—H3A	120.0	N6—C18—C19	124.5 (2)
C4—C3—H3A	120.0	C17—C18—C19	131.9 (2)
C5—C4—C3	118.9 (2)	C18—C19—H19A	109.5
C5—C4—H4	120.6	C18—C19—H19B	109.5
C3—C4—H4	120.6	H19A—C19—H19B	109.5
C4—C5—C6	121.8 (2)	C18—C19—H19C	109.5
C4—C5—H5	119.1	H19A—C19—H19C	109.5
C6—C5—H5	119.1	H19B—C19—H19C	109.5
C1—C6—C5	119.4 (2)	C21—C20—C25	120.5 (3)
C1—C6—H6	120.3	C21—C20—N6	120.2 (2)
C5—C6—H6	120.3	C25—C20—N6	119.3 (2)
N1—C7—N2	111.98 (19)	C20—C21—C22	118.6 (3)
N1—C7—S1	127.76 (17)	C20—C21—H21	120.7
N2—C7—S1	120.26 (16)	C22—C21—H21	120.7
N2—C8—C11	112.53 (19)	C23—C22—C21	121.8 (3)
N2—C8—C9	101.03 (17)	C23—C22—H22	119.1
C11—C8—C9	112.35 (18)	C21—C22—H22	119.1
N2—C8—H8	110.2	C22—C23—C24	117.8 (3)
C11—C8—H8	110.2	C22—C23—C26	121.3 (4)
C9—C8—H8	110.2	C24—C23—C26	121.0 (4)
C10—C9—C8	102.80 (18)	C25—C24—C23	121.1 (3)
C10—C9—H9A	111.2	C25—C24—H24	119.4
C8—C9—H9A	111.2	C23—C24—H24	119.4
C10—C9—H9B	111.2	C24—C25—C20	120.1 (3)
C8—C9—H9B	111.2	C24—C25—H25	119.9
H9A—C9—H9B	109.1	C20—C25—H25	119.9
N3—C10—C17	120.3 (2)	C23—C26—H26A	109.5
N3—C10—C9	114.3 (2)	C23—C26—H26B	109.5
C17—C10—C9	125.3 (2)	H26A—C26—H26B	109.5
C12—C11—C16	118.8 (2)	C23—C26—H26C	109.5
C12—C11—C8	122.7 (2)	H26A—C26—H26C	109.5
C16—C11—C8	118.5 (2)	H26B—C26—H26C	109.5
			
C7—N2—N3—C10	−168.49 (19)	C11—C12—C13—C14	−0.8 (4)
C8—N2—N3—C10	7.2 (2)	C12—C13—C14—C15	−0.5 (6)
C17—N4—N5—N6	−0.1 (2)	C12—C13—C14—F1	179.8 (3)
N4—N5—N6—C18	0.4 (3)	F1—C14—C15—C16	−179.6 (3)
N4—N5—N6—C20	−177.4 (2)	C13—C14—C15—C16	0.8 (7)
C7—N1—C1—C6	−5.6 (4)	C14—C15—C16—C11	0.2 (6)
C7—N1—C1—C2	172.6 (2)	C12—C11—C16—C15	−1.4 (4)
C6—C1—C2—C3	1.1 (3)	C8—C11—C16—C15	175.9 (3)
N1—C1—C2—C3	−177.3 (2)	N5—N4—C17—C18	−0.2 (3)
C1—C2—C3—C4	−0.5 (3)	N5—N4—C17—C10	175.68 (19)
C2—C3—C4—C5	−0.1 (4)	N3—C10—C17—N4	−172.65 (19)
C3—C4—C5—C6	0.3 (4)	C9—C10—C17—N4	3.8 (3)
C2—C1—C6—C5	−0.9 (3)	N3—C10—C17—C18	2.3 (3)
N1—C1—C6—C5	177.3 (2)	C9—C10—C17—C18	178.7 (2)
C4—C5—C6—C1	0.3 (4)	N5—N6—C18—C17	−0.5 (2)
C1—N1—C7—N2	−170.1 (2)	C20—N6—C18—C17	177.1 (2)
C1—N1—C7—S1	9.8 (4)	N5—N6—C18—C19	177.0 (2)
N3—N2—C7—N1	−3.3 (3)	C20—N6—C18—C19	−5.5 (4)
C8—N2—C7—N1	−178.33 (19)	N4—C17—C18—N6	0.4 (2)
N3—N2—C7—S1	176.78 (15)	C10—C17—C18—N6	−175.0 (2)
C8—N2—C7—S1	1.7 (3)	N4—C17—C18—C19	−176.8 (2)
C7—N2—C8—C11	−75.8 (3)	C10—C17—C18—C19	7.8 (4)
N3—N2—C8—C11	108.8 (2)	C18—N6—C20—C21	123.5 (3)
C7—N2—C8—C9	164.2 (2)	N5—N6—C20—C21	−59.1 (3)
N3—N2—C8—C9	−11.2 (2)	C18—N6—C20—C25	−56.8 (4)
N2—C8—C9—C10	10.3 (2)	N5—N6—C20—C25	120.5 (3)
C11—C8—C9—C10	−109.9 (2)	C25—C20—C21—C22	0.9 (4)
N2—N3—C10—C17	177.36 (18)	N6—C20—C21—C22	−179.5 (3)
N2—N3—C10—C9	0.6 (2)	C20—C21—C22—C23	−0.8 (5)
C8—C9—C10—N3	−7.4 (2)	C21—C22—C23—C24	0.5 (5)
C8—C9—C10—C17	175.99 (19)	C21—C22—C23—C26	−179.8 (3)
N2—C8—C11—C12	−18.8 (3)	C22—C23—C24—C25	−0.3 (5)
C9—C8—C11—C12	94.5 (3)	C26—C23—C24—C25	180.0 (3)
N2—C8—C11—C16	164.0 (2)	C23—C24—C25—C20	0.4 (6)
C9—C8—C11—C16	−82.8 (3)	C21—C20—C25—C24	−0.7 (5)
C16—C11—C12—C13	1.7 (4)	N6—C20—C25—C24	179.7 (3)
C8—C11—C12—C13	−175.6 (2)		

### Hydrogen-bond geometry (Å, º)

**Table:**

*D*—H···*A*	*D*—H	H···*A*	*D*···*A*	*D*—H···*A*
N1—H3···N3	0.83 (3)	2.05 (3)	2.568 (3)	120 (2)
